# Associations of serum carotene levels and decline for the ability of attention: a longitudinal study in the Japanese general population

**DOI:** 10.1265/ehpm.25-00090

**Published:** 2025-07-25

**Authors:** Hiroshi Okumiyama, Yoshiki Tsuboi, Ryosuke Fujii, Akihiko Iwahara, Takeshi Hatta, Shuntaro Sato, Hiroya Yamada, Koji Suzuki

**Affiliations:** 1 Department of Preventive Medical Sciences, Fujita Health University School of Medical Sciences, 1-98 Dengakugakubo, Kutsukake-cho, Toyoake, Aichi 470-1192, Japan; 2 Department of Psychology and Collaboration, Kyoto Women’s University Faculty of Psychology and Collaboration, 35 Imagumano, Kitahiyoshi-cho, Higashiyama-ku, Kyoto 605-8501, Japan; 3 Department of Health Sciences, Kansai University of Welfare Sciences Faculty of Health and Welfare, 3-11-1 Asahigaoka, Kashiwara, Osaka 582-0026, Japan; 4 Clinical Research Center, Nagasaki University Hospital, 1-7-1 Sakamoto, Nagasaki 852-8501, Japan; 5 Department of Hygiene, Fujita Health University School of Medicine, 1-98 Dengakugakubo, Kutsukake-cho, Toyoake, Aichi 470-1192, Japan

**Keywords:** Carotenoids, Cognitive decline, Dementia, Oxidative stress

## Abstract

**Background:**

Although serum carotene may contribute to dementia prevention, there is a lack of longitudinal evidence for early cognitive decline before dementia symptoms. The aim of this study was to examine whether serum carotene levels were associated with annually evaluated cognitive trajectories among the Japanese general population.

**Methods:**

Among 581 baseline participants, 199 individuals (83 males; mean age [min, max], 62.7 [39, 90] years) who underwent cognitive assessments more than twice after baseline were analyzed. “Attention” levels were assessed using one- and three-target Digit Cancellation Tests (D-CAT1 and D-CAT3). “General cognitive ability” was assessed by the short version of Mini-Mental State Examination (SMMSE). Serum carotenes (α-carotene, β-carotene and lycopene) were measured by high-performance liquid chromatography. After the measurements, we calculated total carotene levels by summing up the levels of all measured carotene. Carotene levels were categorized into three groups for analysis (low: 0%–25%, middle: 25%–75%, and high: 75%–100%). A linear mixed model was used to estimate the slope of the D-CAT score trajectory and to compare it between three categories.

**Results:**

Compared with the middle carotene group, decline of attention was faster in the D-CAT1 for low β-carotene (*β* = −3.48, *p* = 0.035), lycopene (*β* = −3.10, *p* = 0.062), and total carotene (*β* = −4.75, *p* = 0.003), but not for α-carotene (*β* = −2.60, *p* = 0.111). For the D-CAT3, decline of attention was faster in the group of low lycopene (*β* = −3.17, *p* = 0.002) and total carotene (*β* = −2.17, *p* = 0.037) compared with the middle carotene group, while no clear association for α-carotene (*β* = −0.67, *p* = 0.521) and β-carotene (*β* = −0.64, *p* = 0.639). There were no clear associations between serum carotene and the SMMSE score.

**Conclusions:**

These findings suggest low levels of serum lycopene are associated with a decline of attention in the setting of the general population.

**Supplementary information:**

The online version contains supplementary material available at https://doi.org/10.1265/ehpm.25-00090.

## Graphical Abstract

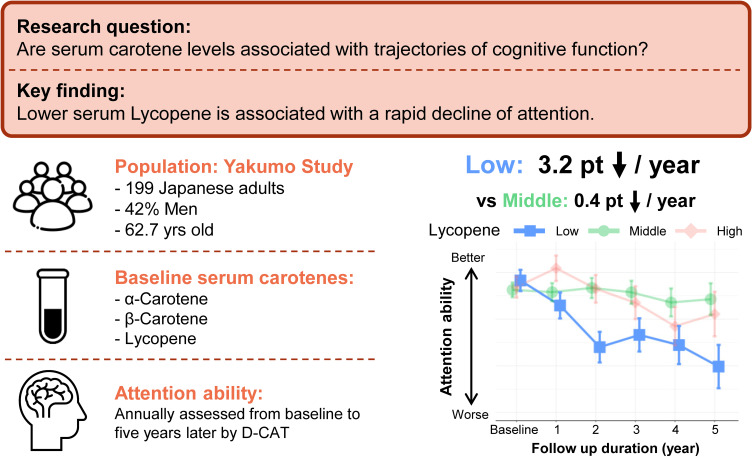

## Introduction

In humans, cognitive function declines as a part of the normal aging process. As cognitive decline accelerates (i.e., the onset of dementia), it begins to affect daily life. With the progression of rapidly aging societies around the world, the number of patients with dementia is also increasing. According to the World Health Organization, approximately 57 million people globally have dementia, with 10 million newly diagnosed patients a year [1]. The higher the number of patients with dementia, the higher the costs for families, communities, and governments. Therefore, this is a problem not only from the perspective of personal health, but also from social and economic viewpoints.

These global trends are evident in Japan. According to the Toyama Dementia Survey, the prevalence of dementia in Japan has been increased over the past 30 years. Furthermore, if this upward trend continues, one in 3.5 people aged 65 or older will have dementia in 25 years [2]. Therefore, in order to halt this upward trend, there is an urgent need to address the prevention of dementia [3]. Several recent studies have reported a relationship between lifestyle-related factors associated with oxidative stress, such as smoking or excess alcohol consumption, and the risk of dementia [4, 5]. Given this situation, lifestyle-related factors known to reduce oxidative stress should receive more attention as nonpharmacologic strategies for dementia prevention.

Carotenes are pigments abundantly found in natural fruits and vegetables [6]. The major carotenes in the human body are α-carotene, β-carotene, and lycopene. These molecules have anti-inflammatory or antioxidative stress properties. Based on evidence from previous studies, carotenes have the potential to reduce the risk of cancer and diseases associated with premature mortality [7, 8]. Furthermore, Craft et al. revealed the existence of these carotenes in the human brain [9]. Therefore, carotenes are expected to have preventive effects against neurodegenerative diseases, including dementia. Numerous studies in animals and humans have reported an association between serum carotene levels and dementia, and a previous meta-analysis found that blood carotene levels were significantly lower in patients with dementia than in cognitive healthy older adults [10]. However, most studies have been reported in cross-sectional designs, and there is a lack of evidence focusing on early cognitive decline before the onset of dementia symptoms. Furthermore, a recent systematic review emphasizes that individual’s cognitive function should be analyzed as a pattern of cognitive decline based on multiple time-point assessments, rather than single assessment [11].

Given this background, in this study, using annually repeated cognitive function test data, we examined whether baseline serum carotene levels were associated with changes and trajectories of individual cognitive function among the Japanese general population.

## Material & methods

**Fig. 1:**
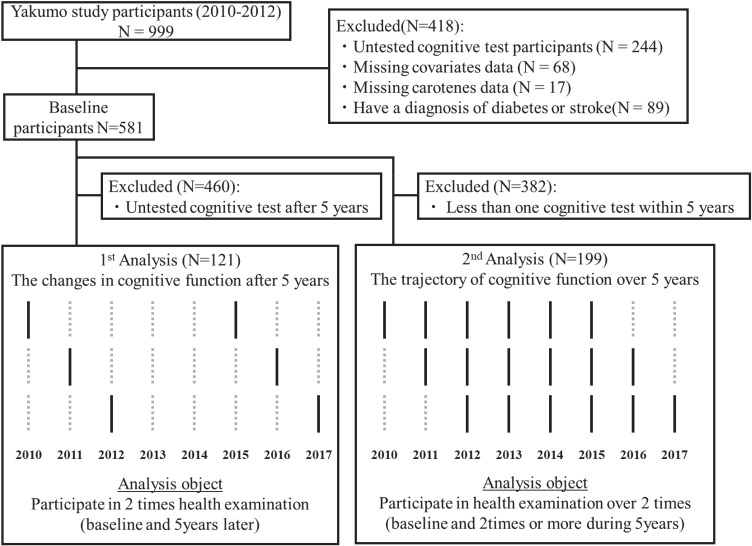
Inclusion criteria and study outline in this study In the conceptual figure for the 1st and 2nd analyses, the solid black lines indicate the year when cognitive testing taken, while the dashed gray lines indicate the year when cognitive testing not taken.

The Yakumo Study is an epidemiological study which performed in Yakumo Town, located in Southern Hokkaido, Japan. The eligible participants of the Yakumo Study are residents who undergo an annual health examination in every August. According to the 2020 Population Census of Japan, the percentage of elderly person (>65 years old) in this town was about 35% [12]. Among a total of 999 people who participated in health examinations in 2010–2012 in Yakumo town, Hokkaido Prefecture, Japan, 581 (220 men and 361 women: mean age [standard deviation (SD)], 62.5 [11.2] years) who had undergone cognitive assessments, serum carotene measurements, and were without any history of stroke or diabetes were included in the study. We performed two longitudinal analyses, as shown in Fig. 1. In the 1st analysis, we investigated the associations between serum carotenes and 5-year changes in cognitive function test scores. Thus, 121 the participants who had undergone a cognitive function test at baseline (2010–2012) and retaken the test 5 years later were included in this analysis. In the 2nd analysis, we focused on the trajectory of an individual’s cognitive function over up to 5 years. This analysis presents the association of carotene with cognitive test scores not only direction (negative or positive) and degree (how much test score decreases) but also speed (how fast per year). In this analysis, 191 participants who had undergone cognitive function tests more than twice after baseline were included. In other words, participants experienced cognitive function tests at least three times (minimum) to six times (maximum) across six-year period (three times: n = 72, four times: n = 43, five times: n = 37, six times: n = 47) and 106 individuals overlapping between 1st and 2nd analysis. Written informed consent was obtained from all participants before the study began. This study was approved by the Ethics Committee of Fujita Health University (approval No. HM24-130).

In this study, to evaluate the early cognitive decline, individuals’ cognitive performances were assessed using two cognitive function tests that have already been validated in Japan [13, 14]. “General cognitive ability” was assessed by the short version of Mini-Mental State Examination (SMMSE). The SMMSE omitted several questions (10 points equivalent) from the original version given that health examination participants can answer those questions correctly. Thus, participants were scored on a range of 0 to 20 points, with the omitted 10 points being added on that. Finally, general cognitive function was assessed between 10 and 30 points. Previous studies reported that SMMSE was comparable to the original version in terms of screening tests and its validity and reliability [13]. Although SMMSE is known as a widespread scale for screening dementia, it has been reported to be less sensitive as a screening tool for early cognitive impairment [15]. To cover the forementioned limitation of SMMSE, “Attention ability”, which is decline in early stage of dementia, was evaluated using the Digit Cancellation Test (D-CAT). This test was developed based on a theory proposed by Sohlberg and Mateer [16]. In this test, participants received a paper printed with randomly distributed numbers and were asked to cross out designated numbers. This test has two tasks: the first is to cross out a target number (D-CAT1) and the second is to cross out three target numbers (D-CAT3). Compared with D-CAT1, D-CAT3 applied more cognitive load because participants are required to keep remembering the multiple target numbers during the test. Total performance, which reflects the ability to focus attention, was analyzed for each test. This index is the total amount of numbers seen by the participants within 1 min, with higher scores indicating higher attention levels.

Blood samples were collected during health examinations, and serum was separated from blood cells within 30 minutes by centrifugation. Serum samples were stored at −80 °C until they were able to be analyzed for carotenes. Serum carotene levels (lycopene, α-carotene, and β-carotene) were measured using high-performance liquid chromatography. The details of the measurements are described elsewhere [17]. After the measurements, we calculated total carotene levels by summing up the levels of all measured carotenes.

Personal information was collected at the study site using a self-administered questionnaire with the support of public health nurses. The information collected included educational attainment (6–9 years, 12 years, 14–16 years), smoking (never, former, and current), alcohol (never, former, and current), exercise (seldom, sometimes, once per week, twice or more per week), and self-reported clinical history of stroke and diabetes (yes or no). Participants who answered “no” were defined as having no history of stroke or diabetes. The body mass index (BMI) was calculated by dividing weight by the square of height based on anthropologic measurements at the health examination site. Biochemical data (alanine amino transferase (ALT) and triglyceride (TG)) were obtained using an auto-analyzer (JCA-BM9130; Nihon Denshi, Tokyo, Japan) at Yakumo General Hospital.

For the analysis of the change of cognitive function test scores, serum carotene levels were log-transformed and standardized for multivariable linear regression. Therefore, β-values calculated in 1st analysis indicates the score change in 5 years for each test per SD difference of baseline serum carotene. For the analysis of the trajectory of cognitive function test scores, all participants were categorized into three groups based on baseline carotene levels: low: 0%–25%, middle: 25%–75%, and high: 75%–100%. Then, we estimated the slope of each individual’s cognitive function level trajectory in the low and high carotene groups, with reference to the middle group, using a linear mixed model. In this model, we implemented a random slope for the participants to allow for an analysis of within-participant correlations of repeated measures over time and random intercepts for variations in baseline cognitive function levels among the participants. The fixed effects included carotene (low, middle, and high), time (0 to 5), and carotene-by-time interaction. To adjust for potential confounders, each model included sex, age at baseline, educational attainment, smoking status, alcohol drinking, exercise, BMI, ALT, TG and cognitive function test score at baseline as covariates. All statistical analyses were conducted using R (version 4.3.0).

## Results

### Study participants’ characteristics

The characteristics of the participants at baseline and each analysis are shown in Table 1. Baseline cognitive function test scores were higher in the 1st or 2nd analysis target than those who were available at baseline. Other covariates were almost similar between baseline eligible participants and 1st or 2nd analysis target. In addition, we described the characteristics of participants who did not include in 1st nor 2nd analysis in Supplementary Table 1. Compared to the participants of 1st and 2nd analysis participants, cognitive function test scores were lower in the participants who were not included in the 1st and 2nd analysis.

**Table 1:** Characteristics of study participants for the baseline (N = 581), 1st (N = 121), and 2nd (N = 199) analysis

	**Baseline** **(N = 581)**	**1st analysis** **(N = 121)**	**2nd analysis** **(N = 199)**
**Women^b^**	361 (62.1)	66 (54.5)	116 (58.3)
**Age, (y)^a^**	62.5 (11.2)	61.3 (8.7)	62.7 (9.3)
**BMI, (kg/m^2^)^a^**	23.6 (3.4)	23.5 (3.0)	23.4 (3.2)
**Education attainment^b^**			
6–9 years	191 (32.9)	29 (24.0)	58 (29.1)
12 years	249 (42.9)	56 (46.3)	87 (43.7)
14–16 years	141 (24.3)	36 (29.8)	54 (27.1)
**Smoking^b^**			
Never	320 (55.1)	67 (55.4)	115 (57.8)
Ever	166 (28.6)	35 (28.9)	59 (29.6)
Current	95 (16.4)	19 (15.7)	25 (12.6)
**Drinking^b^**			
Never	308 (53.0)	61 (50.4)	103 (51.8)
Ever	17 (2.9)	3 (2.5)	4 (2.0)
Current	257 (44.1)	57 (47.1)	92 (46.2)
**Exercise^b^**			
Seldom	336 (57.8)	65 (53.7)	112 (56.3)
Sometimes	108 (18.6)	16 (13.2)	31 (15.6)
Once per week	44 (7.6)	11 (9.1)	17 (8.5)
Twice or more per week	93 (16.0)	29 (24.0)	39 (19.6)
**TG, (mg/dL)^c^**	91.0 [69.0, 125.0]	87.0 [64.0, 121.0]	87.0 [66.0, 119.5]
**ALT, (IU/L)^c^**	21.0 [16.0, 29.0]	20.0 [16.0, 31.0]	21.0 [16.0, 29.5]
**Total carotene, (µM)^c^**	2.20 [1.34, 3.30]	2.20 [1.29, 3.30]	2.25 [1.42, 3.58]
**α-carotene, (µM)^c^**	0.21 [0.13, 0.35]	0.22 [0.12, 0.36]	0.22 [0.13, 0.36]
**β-carotene, (µM)^c^**	1.17 [0.58, 1.84]	1.18 [0.60, 1.89]	1.23 [0.65, 1.99]
**Lycopene, (µM)^c^**	0.72 [0.43, 1.08]	0.71 [0.41, 1.09]	0.71 [0.46, 1.11]
**SMMSE (Point)^a^**	27.8 (2.2)	28.2 (2.1)	28.1 (2.1)
**D-CAT1 (Point)^a^**	270.3 (69.0)	291.7 (66.7)	283.9 (66.6)
**D-CAT3 (Point)^a^**	168.5 (42.0)	177.1 (39.7)	175.6 (40.3)

BMI: Body Mass Index; TG: triglyceride; ALT: alanine aminotransferase, SMMSE; short version of Mini Mental State Examination, D-CAT; digit cancelation test. Baseline participants include 1st and 2nd analysis targets.

^a^Mean (standard deviation) ^b^Number (%) ^c^Median (interquartile range)

### 1st analysis: The change in cognitive function after 5 years

Mean score changes (SD) of all tests after 5 years were SMMSE: 0.46 (2.0), D-CAT1: −8.67 (44.1), and D-CAT3: −4.36 (25.4), respectively. A positive association was found between baseline serum carotene levels and change in D-CAT1 score (total carotene: *β* = 15.3, [CI = 6.0 to 24.7], α-carotene: *β* = 12.1, [CI = 3.7 to 20.4], β-carotene: *β* = 12.8, [CI = 2.8 to 22.9], and lycopene: *β* = 9.8, [CI = 1.6 to 17.9]) (Table 2). Serum total carotene and lycopene were positively associated with changes in D-CAT3 scores (total carotene: *β* = 5.7, [CI = 0.4 to 11.0], lycopene: *β* = 6.1, [CI = 1.6 to 10.6]); however, no associations between serum α-carotene or β-carotene and changes in D-CAT3 scores were observed. In addition, no association between baseline serum carotene levels and the change of score in SMMSE were observed (Supplementary Table 2).

**Table 2:** Association between serum carotene and 5-year change in D-CAT1 and D-CAT3

	**Δ D-CAT1^a^**		**Δ D-CAT3^a^**	

	***β* (95%CI)**	**p-value**	***β* (95%CI)**	**p-value**
**Total carotene**	15.3 (6.0, 24.7)	0.002	5.7 (0.4, 11.0)	0.039
**α-carotene**	12.1 (3.7, 20.4)	0.005	4.4 (−0.4, 9.1)	0.074
**β-carotene**	12.8 (2.8, 22.9)	0.013	3.7 (−1.9, 9.4)	0.199
**Lycopene**	9.8 (1.6, 17.9)	0.020	6.1 (1.6, 10.6)	0.009

D-CAT; digit cancelation test

^a^Δ cognition score = (score at 5 years later − score at baseline)

Adjusted for age at baseline, sex, education attainment, smoking, drinking, exercise, body mass index, triglyceride, alanine aminotransferase, cognitive function test score at baseline.

### 2nd analysis: The trajectory of cognitive function over 5 years

**Fig. 2:**
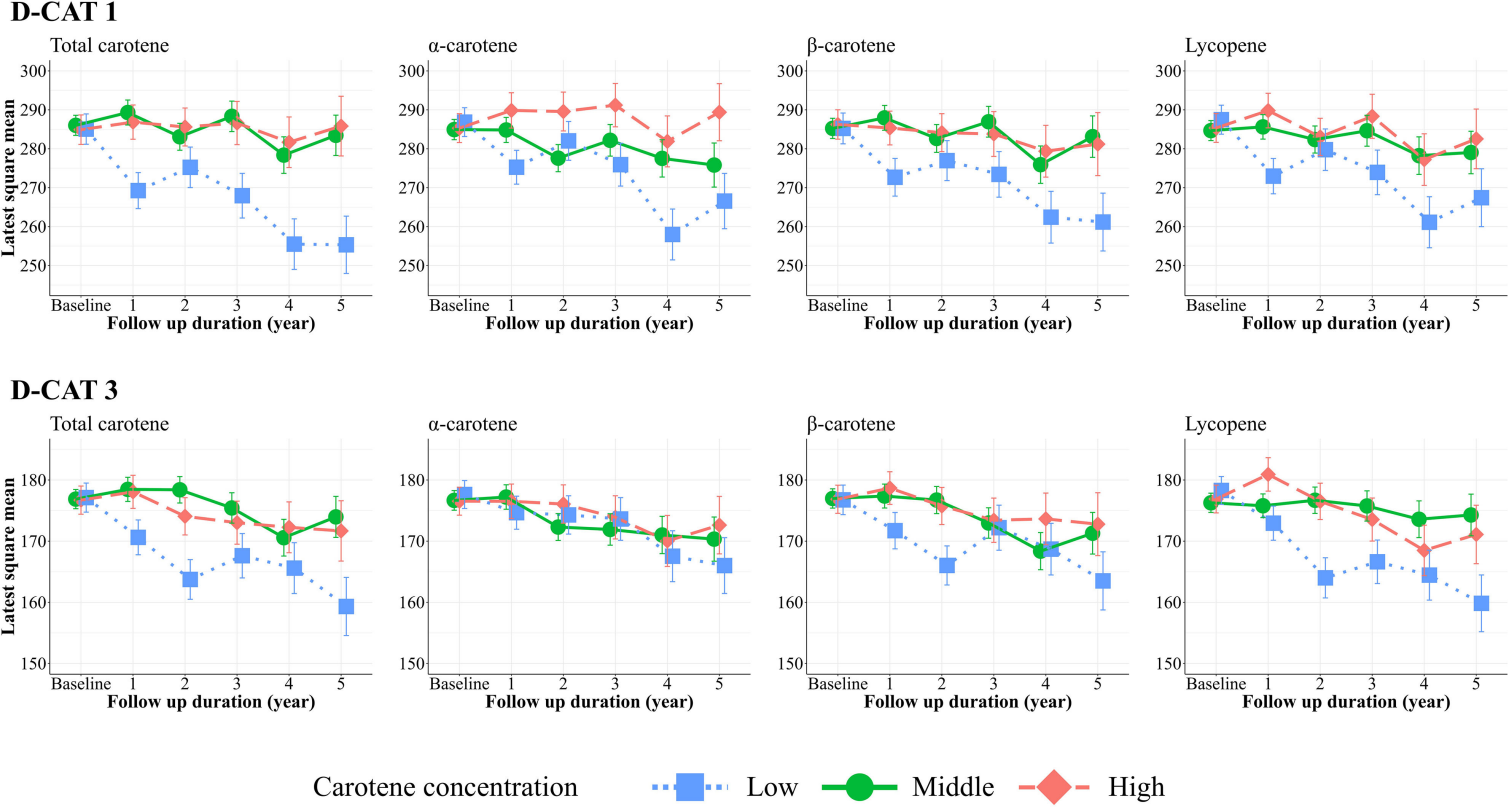
Trajectory of D-CAT1 and D-CAT3 across four carotenes. In each panel, X-axis indicates years during the follow-up period (from baseline to five years), and Y-axis for the least square means for D-CAT1 or D-CAT3 scores. Blue dotted line and square points indicate the low group of carotene levels (<25 percentile), green solid line and circle points for the middle (25–75 percentile), and red long dashed line and diamonds for the high group (>75 percentile). Error bars indicate standard error. The number of participants included in this analysis in each time points were as follows (Baseline: n = 199, 1 year later: n = 150, 2 years later: n = 146, 3 years later: n = 139, 4 years later: n = 115, 5 years later: n = 106)

Compared with the slope of the middle group (green solid line and circle points) for serum total carotene and β-carotene, the low group (blue dotted line and square points) of these carotenes showed a significantly steep slope regarding D-CAT1 scores (total carotene: *β* = −4.76, [CI = −7.91, −1.61], and β-carotene: *β* = −3.48, [CI = −6.68, −0.28]) (Fig. 2, Table 3). The slope tended to be greater for the low than the slope for the middle serum lycopene group (lycopene: *β* = −3.10, [CI = −6.34, 0.15]). However, there is no significant association in the α-carotene (*β* = −2.60, [CI = −5.79, 0.59]). Regarding the D-CAT3, compared with the middle groups (green solid line and circle points) of total carotene and lycopene, the low groups (blue dotted line and square points) showed steep slopes (total carotene: *β* = −2.17, [CI = −4.20, −0.14] and lycopene: *β* = −3.17, [CI = −5.17, −1.17]). However, the slopes in the low groups for α and β-carotene were not as steep as those in the middle groups (α-carotene: *β* = −0.67, [CI = −2.71, 1.39] and β-carotene: *β* = −0.64, [CI = −2.68, 1.42]). Regarding SMMSE, there were no significant associations between baseline serum carotene levels and the slopes of SMMSE score trajectory (Supplementary Fig. 1, Supplementary Table 3).

**Table 3:** Association between serum carotene and the trajectory of D-CAT1 and D-CAT3

	**D-CAT1**		**D-CAT3**	

	***β* (95%CI)**	**p-value**	***β* (95%CI)**	**p-value**
**Total Carotene**				
Time	−1.01 (−2.84, 0.82)	0.282	−0.98 (−2.17, 0.19)	0.104
Interaction terms				
Time × High	0.78 (−2.39, 3.96)	0.632	−0.27 (−2.33, 1.77)	0.794
Time × Middle	Ref	-	Ref	-
Time × Low	−4.76 (−7.91, −1.61)	0.003	−2.17 (−4.20, −0.14)	0.037
**α-carotene**				
Time	−1.86 (−3.79, 0.07)	0.061	−1.52 (−2.76, −0.29)	0.016
Interaction terms				
Time × High	2.04 (−1.18, 5.27)	0.216	0.39 (−1.68, 2.45)	0.713
Time × Middle	Ref	-	Ref	-
Time × Low	−2.60 (−5.79, 0.59)	0.119	−0.67 (−2.71, 1.39)	0.521
**β-carotene**				
Time	−1.07 (−2.94, 0.80)	0.265	−1.56 (−2.76, −0.37)	0.011
Interaction terms				
Time × High	−0.18 (−3.48, 3.10)	0.914	0.50 (−1.60, 2.60)	0.639
Time × Middle	Ref	-	Ref	-
Time × Low	−3.48 (−6.68, −0.28)	0.035	−0.64 (−2.68, 1.42)	0.542
**Lycopene**				
Time	−1.23 (−3.14, 0.69)	0.210	−0.43 (−1.61, 0.75)	0.480
Interaction terms				
Time × High	0.07 (−3.21, 3.35)	0.967	−1.38 (−3.41, 0.64)	0.182
Time × Middle	Ref	-	Ref	-
Time × Low	−3.10 (−6.34, 0.15)	0.062	−3.17 (−5.17, −1.17)	0.002

D-CAT; digit cancelation test

Adjusted for age at baseline, sex, educational attainment, smoking, drinking, exercise, body mass index, triglyceride, alanine aminotransferase, and cognitive function test score at baseline.

## Discussion

In Japanese middle-aged and older adults, we investigated the associations between serum carotenes and longitudinal changes in cognitive function. The findings indicated that lower levels of serum total carotene, α-carotene, and lycopene at baseline were associated with larger decline in attention assessed by the D-CAT among 121 participants, supporting these carotenes may play a role in the decline for the ability of attention. Moreover, a 5-year trajectory-focused analysis indicated that lower levels of serum total carotene and lycopene at baseline were associated with a rapid decline in attention among 199 participants with multi time point cognitive assessment. These findings demonstrate the extent to which these carotenes may contribute to the rate of cognitive decline during the observational period. To the best of our knowledge, this is the first study to investigate the association between serum carotene levels and trajectories regarding individual cognitive function using repeatedly measured data. Although previous longitudinal studies have investigated the associations between serum carotenes and the incidence or mortality of Alzheimer’s disease (AD) [18, 19], the individuals’ cognitive trajectories (how fast or how to decline) might be various as indicated in recent systematic review [11]. Our 2nd analysis addressed this intra-individual variability by capturing longitudinal change through 5-year trajectories and showed that serum lycopene levels were involved in rapid decline of attention levels. Although multiple measurement of cognitive function data might have introduced practice effects that could bias the results, the cognitive function data in our study was measured with approximately 1-year intervals at each time point. Therefore, the practice effect on the association between serum carotene and cognitive trajectory is not considered substantial.

In our analysis, lycopene is associated with decline in D-CAT scores, suggesting that lycopene may contribute to rapid cognitive decline, especially in attention. This finding supports several previous studies in human. Lower lycopene levels are associated with lower scores in neuropsychological tests [20]. Additionally, Devore et al. reported longer consumption is associated with slower cognitive decline [21]. To understand the preventive roles of lycopene in cognitive function, some possible biological mechanisms should be mentioned here. One is reducing oxidative stress, is related to the progression of neurodegenerative diseases such as dementia [22]. Carotene is thought to play an important role in scavenging the oxidative stress generated. In particular, lycopene has the strongest antioxidant capacity which resulted in its molecular structure [23]. Another possible mechanism would be halting some AD pathologies. A recent epidemiological study reported that higher dietary intake of lycopene was associated with the lower severity of Aβ deposition and neuroinflammation in postmortem brain sample [24]. Considering our results with accumulated evidence, lycopene may play a major role in maintaining cognitive health.

However, our findings must be interpreted with caution. Although it has been reported that α- and β-carotene also have antioxidant and anti-inflammatory effects [25, 26], the present results did not show a consistent association between these carotenes and different outcomes (i.e., changes or trajectories). While some studies have reported that α- and β-carotene play a role in dementia prevention, the results have been inconclusive. A recent systematic review focusing on the effects of interventions using β-carotene supplements found no significant evidence regarding a role in preventing cognitive decline [27]. Both α- and β-carotene have beneficial aspects for human health, but few studies have found any preventive effects for carotenes such as lycopene on cognitive function.

In this study, there were no clear associations between serum carotenes and SMMSE scores. The results of the five-year score change analysis indicate that none of the participants in this study showed remarkable changes in their SMMSE scores over the five-year period. This means that the participants in this study do not have significant cognitive decline over the five-year period. Although SMMSE is known as a screening tool for dementia [28], there are several studies indicating its detective limitation for the screening with early-stage of dementia [15, 29]. On the other hand, the great amounts of score changes were found assessed by D-CAT1 or 3. Recent reviews have suggested that the ability of attention can be sensitively impaired in the earliest stages of dementia [30, 31]. Therefore, the results of this study indicate that lycopene may be beneficial in preventing early-stage cognitive decline.

This study has several limitations. First, other major cognitive function domains, such as memory or verbal fluency were not considered in our analysis. Although the ability of attention is known as one of the major domains which decline in the early stage of dementia [30, 31], the association between serum carotene and other cognitive domains were still unclear. In the present study, SMMSE was used as a marker of general cognitive function, but its power to detect early cognitive decline seems to be limited. The use of more detailed cognitive function trajectories of each cognitive domain (e.g., memory or verbal fluency) will allow us to estimate the effects of serum carotene on maintaining overall cognitive function. Second, the results of this study were obtained from health examination participants at a single facility located in a rural area of Japan. Since cognitive decline among the older adults have been reported to be more severe in rural areas than urban areas [32], our findings may not represent the overall Japanese population. Furthermore, the conflicting results between the previous reports and our findings may be due to the differences in sample size or genetic backgrounds related to carotene metabolism [33]. Third, the follow-up rate of this study is not so high. The 1st and 2nd analysis participants’ baseline cognitive functions were higher than that of baseline eligible participants. It may be that the participants who had severe cognitive function at baseline continued to experience cognitive decline and could not retake health examinations in the following years. Therefore, the present results may underestimate the preventive effect of carotene on cognitive decline. Additionally, study designs with fewer dropouts are needed to evaluate the general effects of carotene on cognitive decline prevention. Fourth, our study participants are relatively young compared to the previous work. Therefore, it is possible that differences that would have been observed if the subjects were a bit older were not observed. Because the aim of this study was to investigate the associations between serum carotenes and early cognitive decline, the study participants were relatively young rather than previous reports [18, 19]. However, according to the US cohort, it reported that cognitive decline is already evident in middle age [34]. Although our results may have been limited due to participants’ age, our results were considered in line with our objectives. Fifth, we did not rule out residual confounding, including dietary intake, other antioxidants, and comorbidities. For example, Devore et al. reported that some antioxidants reduced the risk of dementia [35]. Given this, it is required to adjust for vegetable intake or biomarkers in blood. Regarding comorbidities, we excluded participants with major comorbidities such as diabetes and stroke. However, this might be still insufficient for adjusting for other comorbidities. To address these limitations, it is necessary to conduct larger-scale studies with diverse racial and ethnic groups that appropriately account for confounding factors.

In conclusion, lower levels of serum lycopene were associated with rapid declines in attention levels. Although the present results only show the associations between serum lycopene and cognitive domain which is reported in the early stage of dementia, they may imply that avoiding low status of serum lycopene help prevent cognitive decline and dementia.

## Supplementary information

**Additional file 1:** **Supplementary Table 1** Characteristics of study participants not included in 1st and 2nd analysis. **Supplementary Table 2** The results of multiple linear regression between serum carotene and 5-year change in cognitive function test score. **Supplementary Table 3** Regression coefficients from linear mixed models to investigate the associations between serum carotene and the trajectory of cognitive function.

**Additional file 2:** **Supplementary Figure 1:** Trajectory of SMMSE score between three groups (low, middle, and high) across four carotenes.

## Declarations

### Data availability

The data that support the findings of this study are available from the corresponding author upon reasonable request.
