# Supplementary material for: Associations of serum carotene levels and decline for the ability of attention: a longitudinal study in the Japanese general population
Source: Environ Health Prev Med. 2025 Jul 25;30:58. doi: 10.1265/ehpm.25-00090 (PMC12326196; doi:10.1265/ehpm.25-00090)
Supplement: Supplementary file 1 — Additional file 1: Supplementary Table 1 Characteristics of study participants not included in 1st and 2nd analysis. Supplementary Table 2 The results of multiple linear regression between serum carotene and 5-year change in cognitive function test score. Supplementary Table 3 Regression coefficients from linear mixed models to investigate the associations between serum carotene and the trajectory of cognitive function. [file ehpm-30-058-s001.docx]

**Supplementary Table 1** Characteristics of study participants not included in 1st and 2nd analysis

|  | **NOT include 1st nor 2nd** |
| --- | --- |
|  | **(N=367)** |
| **Women ^a^** | 240 (65.4%) |
| **Age, (y) ^a^** | 62.7 (12.1) |
| **BMI, (kg/m2) ^a^** | 23.7 (3.4) |
| **Education attainment ^b^** |  |
| 6–9 years | 130 (35.4%) |
| 12 years | 153 (41.7%) |
| 14–16 years | 84 (22.9%) |
| **Smoking ^b^** |  |
| Never | 199 (54.2%) |
| Ever | 102 (27.8%) |
| Current | 66 (18.0%) |
| **Drinking ^b^** |  |
| Never | 196 (53.4%) |
| Ever | 12 ( 3.3%) |
| Current | 159 (43.3%) |
| **Exercise ^b^** |  |
| Seldom | 215 (58.6%) |
| Sometimes | 75 (20.4%) |
| Once per week | 25 ( 6.8%) |
| Twice or more per week | 52 (14.2%) |
| **TG, (mg/dL) ^c^** | 93.0 [69.0, 126.5] |
| **ALT, (IU/L) ^c^** | 21.0 [16.0, 29.0] |
| **Total carotene, (μM) ^c^** | 2.17 [1.31, 3.17] |
| **α-carotene, (μM) ^c^** | 0.20 [0.13, 0.36] |
| **β-carotene, (μM) ^c^** | 1.15 [0.55, 1.78] |
| **Lycopene, (μM) ^c^** | 0.72 [0.43, 1.06] |
| **SMMSE ^a^ (Point)** | 27.5 (2.2) |
| **D-CAT1 ^a^ (Point/min)** | 263.0 (70.0) |
| **D-CAT3 ^a^ (Point/min)** | 165.3 (42.8) |

BMI: Body Mass Index; TG: triglyceride; ALT: alanine aminotransferase, SMMSE; short version of Mini Mental State Examination, D-CAT; digit cancelation test. Baseline participants include 1st and 2nd analysis targets.

^a^ Mean (standard deviation) ^b^ Number (%) ^c^ Median (interquartile range)

**Supplementary Table 2** The results of multiple linear regression between serum carotene and 5-year change in cognitive function test score

|  | **Δ SMMSE ^a^** | |
| --- | --- | --- |
|  | *β* (95%CI) | p-value |
| **Total carotene** | -0.09 (-0.49 to 0.31) | 0.648 |
| **α-carotene** | 0.04 (-0.31 to 0.40) | 0.818 |
| **β-carotene** | -0.21 (-0.63 to 0.20) | 0.312 |
| **Lycopene** | -0.02 (-0.36 to 0.33) | 0.923 |

SMMSE; short version of Mini Mental State Examination.

^a^ Δ Cognition score = (Score at 5 years later - Score at baseline)

Adjusted for age at baseline, sex, education attainment, smoking, drinking, exercise, body mass index, triglyceride, alanine aminotransferase, cognitive function test score at baseline.

**Supplementary Table 3** Regression coefficients from linear mixed models to investigate the associations between serum carotene and the trajectory of cognitive function.

|  | **SMMSE** | |
| --- | --- | --- |
|  | ***β* (95%CI)** | **p-value** |
| **Total Carotene** |  |  |
| Time | 0.05 (-0.04, 0.15) | 0.297 |
| Interaction |  |  |
| Time × High | 0.03 (-0.14, 0.20) | 0.711 |
| Time × Middle | Ref | - |
| Time × Low | 0.12 (-0.05, 0.28) | 0.165 |
| **α-carotene** |  |  |
| Time | 0.06 (-0.04, 0.16) | 0.230 |
| Interaction |  |  |
| Time × High | 0.05 (-0.12, 0.22) | 0.550 |
| Time × Middle | Ref | - |
| Time × Low | 0.05 (-0.11, 0.22) | 0.535 |
| **β-carotene** |  |  |
| Time | 0.05 (-0.05, 0.15) | 0.315 |
| Interaction |  |  |
| Time × High | 0.04 (-0.13, 0.21) | 0.646 |
| Time × Middle | Ref | - |
| Time × Low | 0.12 (-0.05, 0.28) | 0.162 |
| **Lycopene** |  |  |
| Time | 0.08 (-0.02 to 0.18) | 0.121 |
| Interaction |  |  |
| Time × High | -0.01 (-0.18 to 0.16) | 0.903 |
| Time × Middle | Ref | - |
| Time × Low | 0.05 (-0.11 to 0.22) | 0.539 |

SMMSE; short version of Mini Mental State Examination.

Adjusted for age at baseline, sex, educational attainment, smoking, drinking, exercise, body mass index, triglyceride, alanine aminotransferase, and cognitive function test score at baseline.
