# Supplementary material for: Associations of serum carotene levels and decline for the ability of attention: a longitudinal study in the Japanese general population
Source: Environ Health Prev Med. 2025 Jul 25;30:58. doi: 10.1265/ehpm.25-00090 (PMC12326196; doi:10.1265/ehpm.25-00090)

**Supplementary Figure 1**:**Trajectory of SMMSE score between three groups (low, middle, and high) across four carotenes.** In each panel, X-axis indicates years during the follow-up period (from baseline to five years), and Y-axis for the least square means for SMMSE score. Blue dotted line and square points indicate the low group of carotene levels (<25 percentile), green solid line and circle points for the middle (25-75 percentile), and red long dashed line and diamonds for the high group (>75 percentile). Error bars indicate standard error. The number of participants included in this analysis in each time points were as follows (Baseline: n=199, 1 year later: n=150, 2 years later: n=146, 3 years later: n=139, 4 years later: n=115, 5 years later: n=106 )


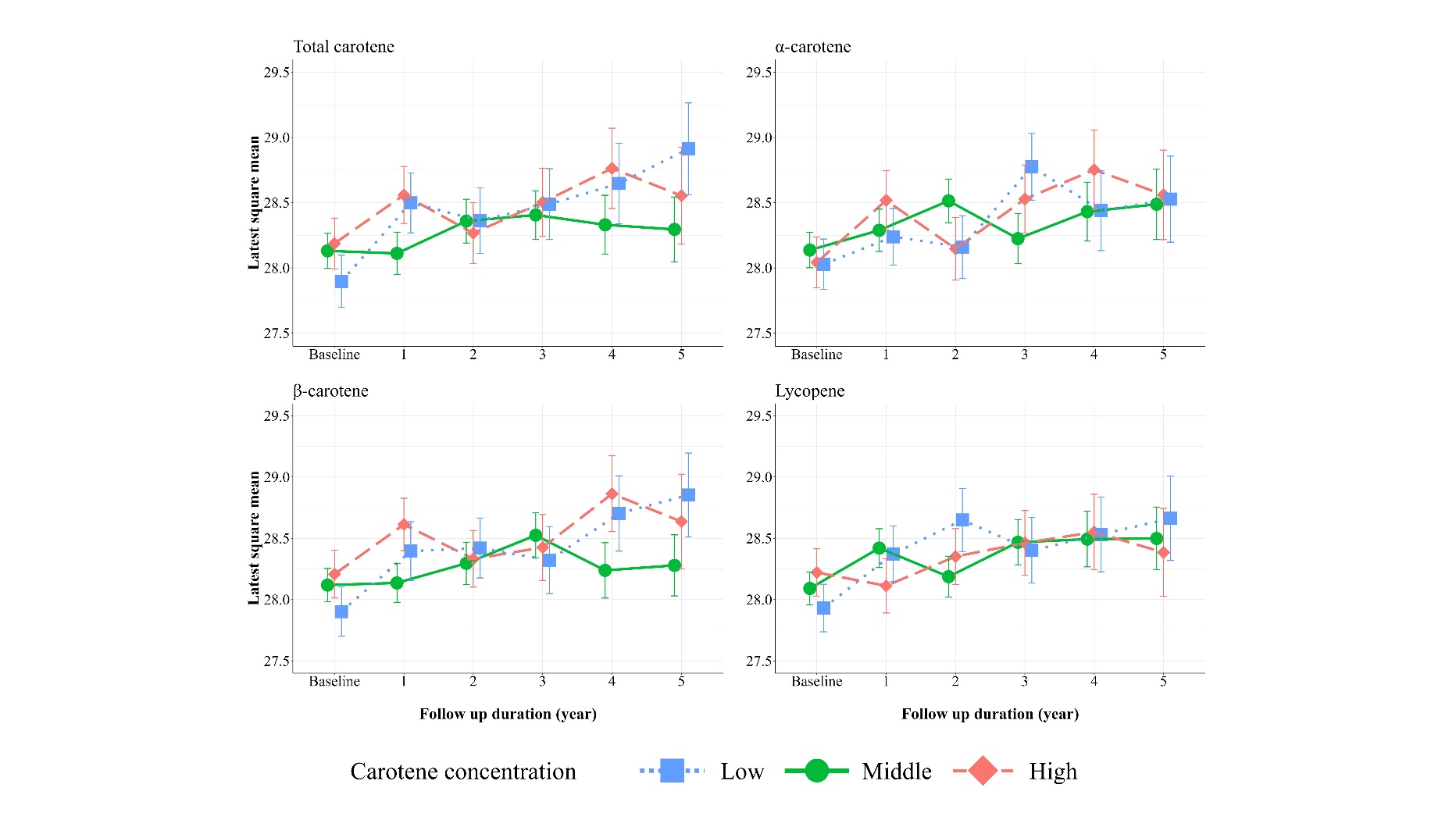

Supplement: Supplementary file 2 — Additional file 2: Supplementary Figure 1: Trajectory of SMMSE score between three groups (low, middle, and high) across four carotenes. [file ehpm-30-058-s002.docx]
